# Non-dipper hypertension is associated with slow coronary flow among hypertensives with normal coronary angiogram

**DOI:** 10.5830/CVJA-2016-045

**Published:** 2017

**Authors:** Ercan Aksit, Erdal Gursul, Fatih Aydin, Murat Samsa, Fatih Ozcelik

**Affiliations:** Department of Cardiology, Biga State Hospital, Canakkale, Turkey; Department of Cardiology, Biga State Hospital, Canakkale, Turkey; Department of Cardiology, Kocaeli State Hospital, Kocaeli, Turkey; Department of Cardiology, Selcuk State Hospital, Izmir, Turkey; Department of Cardiology, Trakya University Hospital, Edirne, Turkey

**Keywords:** hypertension, coronary angiography, TIMI frame count, dipper, non-dipper

## Abstract

**Aim:**

A person with a drop of more than 10% in nocturnal arterial blood pressure during the circadian rhythm is referred to as a dipper and one with a smaller decrease is referred to as a non-dipper. In our study, we aimed to compare the thrombolysis in myocardial infarction (TIMI) frame count in non-dipper and dipper hypertensive patient groups who had normal coronary artery angiography

**Methods:**

Patients with normal coronary arteries and with ambulatory blood pressure monitoring follow ups were retrospectively investigated and 60 patients (35%, female) were included in our study. The patients were grouped as dipper (n = 30) and non-dipper (n = 30) hypertensives.

**Results:**

The TIMI frame counts in all three coronary arteries and the mean TIMI frame count in the dipper hypertensive patient group were significantly lower than those of the non-dipper hypertensives (right coronary artery TIMI frame count: 16.83 ± 3.70; 21.63 ± 3.44, p < 0.001; circumflex artery TIMI frame count: 21.28 ± 3.52; 25.65 ± 3.61, p < 0.001; left anterior descending artery TIMI frame count: 34.20 ± 2.80; 37.05 ± 3.30, p = 0.001; corrected left anterior descending artery TIMI frame count: 20.05 ± 1.63; 21.74 ± 1.95, p = 0.001; mean TIMI frame count: 19.31 ± 2.3; 22.94 ± 2.61, p < 0.001). The body mass index (BMI) was 23.79 ± 2.81 kg/m^2^ in the dipper patient group, while it was 25.47 ± 2.92 in the non-dippers. BMI was found to be significantly higher in the non-dipper group than in the dipper group (p = 0.027).

**Conclusions:**

In this study, TIMI frame count, which is a simple, productive, objective and reproducible method for determination of microvascular changes, was found to be higher in non-dipper hypertensive patients than in the dipper patients.

## Aim

Hypertension is a significant risk factor for stroke, myocardial infarction, renal diseases and other vascular disorders. Treatment of high blood pressure may lower the incidence of complications and enable a longer life. Cardiovacular parameters such as blood pressure, heart rate and coronary tonus change with the daily circadian rhythm.^1^

Development of ambulatory blood pressure-monitoring (ABPM) has provided an understanding of diurnal blood pressure variations.^2^ According to ABPM data obtained from healthy subjects, blood pressure reaches its highest levels in the morning, decreases slowly during the day and maintains lowest levels during the night.^3^ This circadian rhythm in blood pressure has led to a novel classification. In this ABPM-dependent classificiation, if nocturnal blood pressure decreases more than 10% of the day-time levels, it is called dipper hypertension and if the drop is less than 10%, it is considered non-dipper hypertension.^4^

The mechanism of diurnal blood pressure variation disorders is not clear. At night, the balance in the autonomous nervous system probably shifts towards the sympathetic nervous system.^5^ If the blood pressure decrease is less than 10 to 20% during sleep, it is connected with target-organ damage. Particularly in non-dipper hypertensive patients, it is common to see left ventricular hypertrophy, congestive heart failure, myocardial infarction, stroke and renal failure (albuminuria and end-stage renal failure).^6^,^7^

The thrombolysis in myocardial infarction (TIMI) frame count is the sum of the ciné-angiographic squares obtained, after infusing opaque substance into the coronaries, from the time when the dye is seen at the level of the coronary artery ostium to when it reaches the distal part. The TIMI frame count, which is a simple, objective and reproducible method, is a quantitative predictor of coronary flow rate. A high TIMI frame count is a predictor of slow coronary flow and endothelial dysfunction.^8^

The aim of this study was to compare the TIMI frame count in dipper and non-dipper hypertensive patients with normal coronary artery angiography (CAG).

## Methods

This retrospective, single-centre study was performed in a tertiary healthcare centre. The study data were obtained between 15 February 2010 and 15 February 2012 from hypertensive patients aged between 18 and 80 years who had normal CAG and who had arterial blood pressure follow up with ABPM. Patients who were below 18 and above 80 years, with coronary artery disease, haemodynamic changes, congenital heart disease, left ventricular ejection fraction below 55%, and those who had severe valve disease were excluded. The study was approved by the Trakya University Ethics Board (08.02.2012, Decision Nr.05/12, Protocol Nr. 201/39).

Age, gender, height, weight, hypertension and diabetes mellitus status, smoking and alcohol consumption, medications, electrocardiogram (ECG), lipid profile and creatinine levels were obtained from the patient files. Body mass index (BMI) was calculated by dividing the body weight (kg) by the square of the height (m). Echocardiogram (echo) reports were obtained from the echo laboratory, coronary artery images from the CAG laboratory, and 24-hour blood pressure levels were obtained from the effort-Holter laboratory.

The echo evaluations of the patients were performed using the Vivid 7 Pro (General Electric Medical Systems, Milwaukee, Wisconsin). Left ventricular ejection fraction (EF) was calculated with the M-mode imaging method.

Ambulatory blood pressure follow up was done with the tension artery Holter device (DMS 300-3A Holter Recorder) register system using the ambulatory blood pressure Spacelab 90207 device (Space Labs Inc, Richmond, Washington, USA). ABPM was performed before hospitalisation, irrespective of the type and duration of antihypertensive drug therapy. The patients were recommended to maintain daily activities and to hold the arms straight during measurement. Blood measurements were made every 15 minutes between 07:00 and 23:00, and every 20 minutes between 23:00 and 07:00. Using short time intervals, the 10:00–22:00 interval was accepted as the day-time, and the 24:00–06:00 interval as the night-time period. The recorded data were evaluated at the end of 24 hours.

Systolic and diastolic blood pressure levels and heart rate measurements were evaluated for the day and night-time periods. If the mean systolic and diastolic blood pressure levels decreased by less than 10% or did not fall, the patient was considered a non-dipper, and if it decreased by more than 10%, it was considered dipper hypertension.^9^,^10^

All of the patients had undergone CAG due to a positive myocardial perfusion ischaemia test. The coronary angiography procedures had been performed up to one month after the ABPM. Coronary artery evaluation of the patients was made using the Philips Integris H 3000 (Eindhoven, The Netherlands) angiogram device. The presence or absence of coronary artery disorders was recorded. Patients who had any degree of coronary artery stenosis or plaques and those who had haemodynamic changes that may have affected the square counts during the angiogram were excluded. The TIMI frame count of patients whose coronary arteries were normal was separately calculated for all three coronary arteries. Nitrate was not administered to any patients during the CAG, as it could have affected the results of the measurements.

Gibson et al. were the first to present the TIMI frame count, or the TIMI square-count method as a simple, productive, objective and quantitative technique to provide a standard index for coronary blood flow measurement. They investigated the angiographic images of the TIMI-4 study.^8^

The TIMI frame count was calculated by an independent operator (always the same operator), who did not know the AMBP results. To determine the TIMI frame count following administration of the opaque material, the ciné-angiographic square counts seen between the level of the stained coronary artery ostium and its distal part were added up. The first square was taken at the moment when an opacification was seen filling the whole of the coronary artery orifice and moving forward.

The last square was caught at the moment when the dye reached the standard marker point determined separately for the three arteries by Gibson et al. at the distal part of the vessel.8 The prediction points were the distal bifurcation branching point for the left anterior descending artery (LAD) (whale-tail sign at bifurcation point), the branchlet separating from the distal bifurcation at the furtherest point where the artery opacified after the lesion for the circumflex artery (Cx), and the filling moment of the posterolateral branchlet after the posterior descending artery for the right coronary artery (RCA). The best projection angle was the right anterior oblique or the left anterior oblique-caudal angle for the LAD and Cx, and the left anterior oblique-cranial angle for the RCA.

In Gibson and co-workers’ study, the TIMI frame count for the RCA was 20.4 ± 3.0, the square count for the Cx artery was 22.2 ± 4.1, and for the LAD, it was 36.2 ± 2.6 (p < 0.001).^8^ These values were standard measurements when the coronary angiogram device could take 30 frames/s. If the coronary angiogram worked at a rate of 12.5 frame/s, to adjust to standard measurements, the value was multiplied by 2.4. If the coronary angiogram worked at a rate of 25 frame/s, to adjust to standard measurements, the value was multiplied by 1.2. If the coronary angiogram worked at a rate of 15 frame/s, to adjust to standard measurements, the value was multiplied by two.

For the LAD, the correction for this difference was made by dividing the square count by 1.7. Therefore, the TIMI square count corrected for the LAD (cLAD) was determined as 21.1 ± 1.5 squares. The mean TIMI frame count was calculated by adding three coronary artery TIMI frame counts and dividing by three.

## Statistical analysis

For the descriptive statistics of the data, the mean, standard deviation and ratios were used. The Kolmogorov–Smirnov test was used for distribution of the data. Comparison of the means between the two groups was done using the independent samples t-test. The Chi-squared test was used for analysis of the ratios. The SPSS 20.0 program was used for the analysis. Statistical significance was set at p < 0.05 for all analyses.

## Results

Sixty patients (38% female), whose mean age was 52.85 ± 10.42 years, were included in the study. The demographic and clinical data of the patients are presented in Table 1. The patients were grouped as dippers (n = 30) and non-dippers (n = 30). The demographic and clinical data of the groups were compared. No significant differences were found between gender, age, smoking and alcohol consumption of the dipper and non-dipper patient groups.

**Table 1:** Distribution of patients based on demographic and clinical characteristics

*Demographic and clinical parameters*	*Patients (n = 60)*
Age (year ± SD)	52.85 ± 10.42
Female, n (%)	23 (38.3)
BMI (kg/m^2^ ± SD)	24.63 ± 2.8
Smoking, n (%)	32 (53.3)
Drinking, n (%)	29 (48.3)
Hyperlipidaemia, n (%)	27 (45.0)
Diabetes mellitus n (%)	8 (13)
Antihypertensive drugs	
ACEI, n (%)	21 (35.0)
ARB, n (%)	20 (33.3)
BB, n (%)	24 (40.0)
CCB, n (%)	22 (36.6)
HDL-C (mg/dl ± SD)	47.63 ± 13.21
(mmol/l)	1.23 ± 0.34
LDL-C (mg/dl ± SD)	133.55 ± 40.91
(mmol/l)	3.46 ± 1.06
FPG (mg/dl ± SD)	95.82 ± 15.04
(mmol/l)	5.32 ± 0.83
TG (mg/dl ± SD)	132.16 ± 74.34
(mmol/l)	1.49 ± 0.84
Total cholesterol (g/dl ± SD)	185.16 ± 42.7
(mmol/l)	4.80 ± 1.11
Creatinine (mg/dl ± SD)	0.79 ± 0.18
(mmol/l)	69.84 ± 15.91
Haemoglobin (g/dl ± SD)	13.6 ± 1.61
Ejection fraction (% ± SD)	66.61 ± 3.8

Continuous data are expressed as mean ± SD, categorical data are expressed as n (%). SD: standard deviation, ACEI: angiotensin converting enzyme inhibitors, ARB: angiotensin receptor blocker, BB: beta-blocker, CCB: calcium channel blockers, HDL-C: high-density lipoprotein cholesterol, LDL-C: low-density lipoprotein cholesterol, FPG: fasting plasma glucose, TG: triglycerides.

The BMI in the non-dipper group (25.47 ± 2.92 kg/m^2^) was significantly higher than that of the dippers (23.79 ± 2.81 kg/m^2^) (p = 0.027). There was no significant difference between symptoms, hyperlipidaemia and diabetes mellitus between the dipper and non-dipper groups. Similarly, the use of angiotensin converting enzyme inhibitors (ACEI), angiotensin receptor blockers (ARB), beta-blocker (BB) and calcium channel blockers (CCB) did not demonstrate any significant dfferences between the groups. Of the laboratory parameters, no statistically significant difference was found between high-density lipoprotein (HDL-C) cholesterol, low-density lipoprotein (LDL-C) cholesterol, fasting blood glucose (FBG), triglyceride (TG), total cholesterol (TC), creatinine, haemoglobin and ejection fraction (EF) levels (Table 2). None of the patients were on nitrate treatment.

**Table 2:** Comparison of the demographic and clinical characteristics between the groups

**	*Dipper group*	*Non-dipper group*	**
*Demographic and clinical parameters*	*(n = 30)*	*(n = 30)*	*p-value*
Age (year ± SD)	51.63 ± 12.68	54.07 ± 8.17	0.381*
Gender			
Male, n (%)	16 (53.3)	21 (70.0)	0.184#
Female, n (%)	14 (46.7)	9 (30.0)	
BMI (kg/m^2^ ± SD)	23.79 ± 2.81	25.47 ± 2.92	0.027*
Smoking, n (%)	14 (46.7)	18 (60.0)	0.301^#^
Drinking, n (%)	11 (36.7)	8 (26.7)	0.405^#^
Hyperlipidaemia, n (%)	13 (43.3)	14 (46.7)	0.895^#^
Diabetes mellitus, n (%)	3 (10.0)	5 (16.7)	0.448^#^
Symptoms			
Chest pain, n (%)	30 (100)	30 (100)	1^#^
Palpitation, n (%)	14 (46.6)	15 (50)	0.823^#^
Dsypnoea, n (%)	9 (30.0)	10 (33.3)	0.437^#^
Restlessness, n (%)	8 (26.6)	14 (46.6)	0.248^#^
Dizziness, n (%)	8 (26.6)	12 (40.0)	0.312#
Antihypertensive drugs			
ACEI, n (%)	11 (36.7)	10 (33.3)	0.737^#^
ARB, n (%)	10 (33.3)	10 (33.3)	1^#^
CCB, n (%)	9 (30.0)	13 (43.3)	0.234^#^
BB, n (%)	12 (40.0)	12 (40.0)	1^#^
Statin, n (%)	9 (30.0)	13 (43.3)	0.234^#^
HDL-C (mg/dl ± SD)	47.23 ± 13.51	48.03 ± 12.91	0.815*
(mmol/l)	(1.22 ± 0.35)	(1.24 ± 0.33)	
LDL-C (mg/dl ± SD)	135.55 ± 42.16	131.56 ± 39.67	0.707*
(mmol/l)	(3.51 ± 1.09)	(3.41 ± 1.03)	
FPG (mg/dl ± SD)	93.97 ± 15.89	97.63 ± 14.22	0.350*
(mmol/l)	(5.22 ± 0.88)	(5.42 ± 0.79)	
TG (g/dl ± SD)	114.50 ± 59.67	149.83 ± 89.09	0.076*
(mmol/l)	(1.29 ± 0.67)	(1.69 ± 1.01)	
Total cholesterol (g/dl ± SD)	188.00 ± 44.08	182.33 ± 41.47	0.610*
(mmol/l)	(4.87 ± 1.14)	(4.72 ± 1.07)	
Creatinine (mg/dl ± SD)	0.81 ± 0.19	0.77 ± 0.18	0.404*
(mmol/l)	(71.60 ± 16.80)	(68.07 ± 15.91)	
Haemoglobin (g/dl ± SD)	13.56 ± 1.82	13.70 ± 1.41	0.728*
Ejection fraction (% ± SD)	66.40 ± 4.10	66.83 ± 3.61	0.666*

Continuous data are expressed as mean ± SD, categorical data are expressed as n (%).

*Chi-squared test, #Independent samples t-test, statistical significance level is p < 0.05 (bold values).

SD: standard deviation, ACEI: Angiotensin converting enzyme inhibitors, ARB: angiotensin receptor blocker, BB: beta-blocker, CCB: calcium channel blockers, HDL-C: high-density lipoprotein cholesterol, LDL-C: low-density lipoprotein cholesterol, FPG: fasting plasma glucose, TG: triglycerides.

The 24-hour Holter data of the patients were evaluated and inter-group comparisons were performed. In the dipper group, the pulse rate (66.57 ± 4.92 bpm) was significantly lower than that of the non-dipper group (72.70 ± 4.86 bpm) (p = 0.001). No statistically significant differences were determined between the groups with regard to the mean systolic and mean diastolic blood pressures (p = 0.226, p = 0.749, respectively). Similarly, there was no significant difference between the groups regarding the day-time mean systolic and diastolic blood pressures (p = 0.802, p = 0.417, respectively). The night-time mean systolic and diastolic blood pressure levels were, however, significantly lower in the dipper group (p = 0.001, p ≤ 0.001, respectively). The percentage change in systolic and diastolic blood pressures was significantly higher in the dipper than the non-dipper group (p ≤ 0.001, p ≤ 0.001, respectively). The inter-group comparisons of ABPM are presented in Table 3.

**Table 3:** Comparison of ambulatory blood pressure data between the groups

**	*Dipper group*	*Non-dipper group*	**
*Ambulatory blood pressure*	*(n = 30)*	*(n = 30)*	p-value*
Pulse (bpm ± SD)	66.57 ± 4.92	72.70 ± 4.86	0.001
Over 24 hours			
Mean systolic BP (mmHg ± SD)	118.93 ± 14.8	124.37 ± 19.23	0.226
Mean diastolic BP (mmHg ± SD)	68.76 ± 10.55	69.53 ± 8.64	0.749
Day-time			
Mean systolic BP (mmHg ± SD)	123.47 ± 16.67	124.63 ± 19.08	0.802
Mean diastolic BP (mmHg ± SD)	71.87 ± 11.49	69.67 ± 9.21	0.417
Night-time			
Mean systolic BP (mmHg ± SD)	108.10 ± 13.61	123.50 ± 20.49	0.001
Mean diastolic BP (mmHg ± SD)	60.60 ± 8.94	69.13 ± 8.3	< 0.001
Systolic BP variation (% ± SD)	13.33 ± 4.47	1.32 ± 5.49	< 0.001
Diastolic BP variation (% ± SD)	15.57 ± 5.74	2.03 ± 6.03	< 0.001

Continuous data are expressed as mean ± SD.

*Independent samples t-test, statistical significance level is p < 0.05 (bold values)

BPM: beat per minute, SD: standard deviation, BP: blood pressure.

The mean TIMI frame counts of all three coronary arteries were calculated in the dipper and non-dipper patient groups. In the dipper group, the RCA TIMI frame count (16.83 ± 3.70) was significantly lower than that in the non-dipper group (21.63 ± 3.44) (p < 0.001). In the dipper group, the Cx TIMI frame count (21.28 ± 3.52) was significantly lower than in the non-dipper group (25.65 ± 3.61) (p < 0.001). The LAD TIMI frame count in the dipper group (34.20 ± 2.80) was significantly lower than in the non-dipper group (37.05 ± 3.30) (p = 0.001). The LAD corrected TIMI frame count in the dipper group (20.05 ± 1.63) was significantly lower than in the non-dipper group (21.74 ± 1.95) (p = 0.001). In the dipper group, the mean TIMI frame count (19.31 ± 2.31) was significantly lower than in the non-dipper group (22.94 ± 2.61) (p < 0.001) (Table 4).

**Table 4:** Comparison of TIMI frame scores between the groups

**	*Dipper group*	*Non-dipper group*	**
*TIMI fram scores*	*(n = 30)*	*(n = 30)*	p-value*
RCA TIMI frame score	16.83 ± 3.70	21.63 ± 3.44	< 0.001
Cx TIMI frame score	21.28 ± 3.52	25.65 ± 3.61	< 0.001
LAD TIMI frame score	34.20 ± 2.80	37.05 ± 3.30	0.001
LAD corrected TIMI frame score	20.05 ± 1.63	21.74 ± 1.95	0.001
The average TIMI frame score	19.31 ± 2.31	22.94 ± 2.61	< 0.001

Continuous data are expressed as mean ± SD.

*Chi-squared test, statistical significance level is p < 0.05 (bold values).

RCA: right coronary artery, TIMI: thrombolysis in myocardial infarction, Cx: circumflex artery, LAD: left anterior descending artery.

## Discussion

Arterial blood pressure has a daily circadian rhythm. Physiologically, nocturnal blood pressure decreases by more than 10% compared to day-time levels, and this is called dipper hypertension. If the nocturnal blood pressure has a less than 10% fall from day-time blood pressure values, it is considered as non-dipper hypertension.^10^ The reason for such classification is due to the differences in morbidity and mortality rates between these groups. In patients who have non-dipper blood pressure, end-organ damage (ventricular hypertrophy, microalbuminuria, decreased arterial compliance) as well as cardiovascular morbidity and mortality rates are higher.^11^,^12^

In order to develop a standard index for coronary blood flow measurement, Gibson et al. presented the TIMI frame count as a simple, productive, objective and quantitative technique by investigating the angiographic images of the TIMI-4 study.^8^ After administration of an opaque material, the TIMI square count is the sum of the ciné-angiographic squares seen between the level of the stained coronary artery ostium and its distal part on CAG. A high TIMI frame count is related to a slow flow rate and endothelial dysfunction.^13^ In our study, we compared the TIMI frame count in dipper and non-dipper hypertensive patient groups who had normal CAG.

According to a study by Yazici et al., the number of non-dipper patients was significantly higher than dipper patients in a patient group with slow coronary flow rates. In that study, the non-dipper patients with slow coronary flow rates had a higher percentage of unstable angina-like features, recurrent chest pain, frequency of malignant ventricular arrhythmia and sudden cardiac death rates than dipper patients.^14^

Evola et al. compared the TIMI frame counts of 80 hypertensive patients with normal CAG with 15 normotensive subjects, and found higher TIMI scores in the hypertensive group. In the same study, when the hypertensive patients with negative and positive myocardial perfusion scintigraphy were compared, the TIMI frame counts were significantly higher in patients with positive scintigraphy.^15^ From these data, they predicted that coronary artery flow and myocardial perfusion disorders were more frequent in the group with high TIMI frame counts. They concluded that myocardial perfusion scintigraphy could be used as a non-invasive diagnostic test to determine early changes in coronary microcirculation.

In our study, we found a higher TIMI frame count in all three coronary arteries in the non-dipper hypertensive patient group compared to the dipper group. In their study showing the significance of small-vessel disorder, Pekdemir et al. investigated the coronary anatomy using intravascular ultrasonography (IVUS) and epicardial resistance with fractional flow reserve (FFR).^16^ They stated that in patients with slow coronary flow, the increase in resistance in epicardial coronary arteries could play a role in the development of early diffuse atherosclerosis.

In a patient group with slow coronary flow, Xia et al. discovered higher serum uric acid, platelet count, high-sensitivity C-reactive protein (CRP) and two-hour fasting glucose levels compared to the control group.^17^ In recent epidemiological and experimental studies, a high uric acid level has been proven to be a cardiovascular risk factor.^18^,^19^

Using the TIMI frame count, Turhan et al. compared coronary blood flow in 42 metabolic syndrome patients and a control group of 42 subjects without the metabolic syndrome. The TIMI frame count was statistically significantly higher in patients with higher values of waist circumference, body mass index and triglyceride levels.^20^ In our study, the body mass index was statistically significantly higher in the non-dipper group than in the dipper group.

According to numerous robust evidence, deterioration of endothelial-dependent vasodilatation as a result of a decrease in nitric oxide release in brachial, coronary, renal and small arteries is a risk factor in cardiovascular and cerebrovascular patients.^21^-^26^ Higashi et al. compared endothelial dysfunction in 20 non-dipper and 20 dipper hypertensive patients.^27^ The endothelial dysfunction predictors were decreased nitric oxide final products, nitrite/nitrate and cyclic guanicine monophosphate in 24-hour urine samples. In the non-dipper patient group, nitrite/nitrate and cyclic guanicine monophosphate levels in 24-hour urine samples were statistically significantly lower. If we consider the TIMI frame count as a predictor of endothelial dysfunction, finding a higher TIMI frame count in all three coronary arteries confirms the study by Higashi et al.^27^

The most important limitations of our study were its retrospective design and the small sample size, as well as the lack of testing for other biochemical and echocardiographic markers that have shown a relationship with coronary slow flow. Studies with a prospective design integrating a larger number of patients and coronary slow-flow markers would provide more valuable data. In our study, the proximal coronary artery diameters were not compared. Due to the fact that vasoconstriction, which may develop secondarily due to an increase in sympathetic tone, may have had an effect on the TIMI frame count. Measurement of proximal artery diameters and comparing them between the two groups would produce more valuable information.

In our study, we found higher TIMI frame counts in all three coronary arteries and a higher mean TIMI frame count in the non-dipper hypertensive patients than in the dipper group. Microvascular bed changes and endothelial dysfunction in non-dipper hypertensive patients can be confirmed with TIMI frame count, which is a predictor of coronary slow flow rate.

## Conclusion

In this study, the TIMI frame count, which is a simple, productive, objective and reproducible method for indirect determination of microvascular changes, was found to be higher in non-dipper hypertensive patients than in dipper hypertensives.

## References

[1] Millar-Craig MW, Bishop CN, Raftery EB (1978). Circadian variation of blood-pressure.. Lancet.

[2] Pickering TG (1990). The clinical significance of diurnal blood pressure variations: dippers and nondippers.. Circulation.

[3] Seo WS, Oh HS (2002). The circadian rhythms of blood pressure and heart rate in the hypertensive subjects: dippers and non-dippers.. Yonsei Med J.

[4] Fujii T, Nishimura M (1999). Circadian rhythm of natriuresis is disturbed in nondipper type of essential hypertension.. Am J Kidney Dis.

[5] Kurpesa M, Trzos E, Drozdz J (2002). Myocardial ischemia and autonomic activity in dippers and non-dippers with coronary artery disease: assessment of normotensive and hypertensive patients.. Int J Cardiol.

[6] Kobrin I, Oigman W, Kumar A (1984). Diurnal variation of blood pressure in elderly patients with essential hypertension. J Am Geriatr Soc.

[7] Shimada K, Kawamoto A, Matsubayashi K (1992). silent cerebrovasculer damage in elderly patients with hypertension.. J Hypertens.

[8] Gibson CM, Cannon CP, Daley WL (1996). TIMI frame count: a quantitative method of assessing coronary artery flow.. Circulation.

[9] Roman MJ, Pickering TG, Schwartz JE (1997). Is the absence of a normal nocturnal fall in blood pressure (nondipping) associated with cardiovascular target organ damage?. J Hypertens.

[10] O'Brien E, Sheridan J, O'Malley K (1988). Dippers and non-dippers. Lancet.

[11] Ohkubo T, Hozawa a, Yamaguchi J (2002). Prognostic significance of the nocturnal decline in blood pressure in individuals with and without high 24-h blood pressure: The Ohasoma study. J Hypertens.

[12] Vardecchia P, Schilacci G, Borgioni C (1997). Altered circadian blood pressure profile and prognosis.. Blood Pressure Monitor.

[13] Cushman WC, Ford CE, Einhorn PT (2008). Blood pressure control by drug group in the Antihypertensive and Lipid-Lowering Treatment to Prevent Heart Attack Trial (ALLHAT).. J Clin Hypertens (Greenwich).

[14] Yazıcı M, Demircan S, Durna K (2005). The incidence of nondipping state in normotensive patients with coronary slow flow and its relationship with prognosis.. Arch Turk Soc Cardiol.

[15] Evola S, Cuttitta F, Evola G (2012). Early detection of coronary artery flow and myocardial perfusion impairment in hypertensive patients evidenced by Myocardial Blush Grade (MBG) and Thrombolysis in Myocardial Infarction (TIMI) Frame Count (TFC).. Intern Med.

[16] Pekdemir H, Cin VG, Cicek D (2004). Slow coronary flow may be a sign of diffuse atherosclerosis. Contribution of FFR and IVUS.. Acta Cardiol.

[17] Xia S, Deng SB, Wang Y (2010). Clinical analysis of the risk factors of slow coronary flow. Heart Vessels.

[18] Niskanen LK, Laaksonen DE, Nyyssonen K (2004). Uric acid level as a risk factor for cardiovascular and all-cause mortality in middle-aged men: a prospective cohort study.. Arch Intern Med.

[19] Ioachimescu AG, Brennan DM, Hoar BM (2008). Serum uric acid is an independent predictor of all-cause mortality in patients at high risk of cardiovascular disease: a preventive cardiology information system (PreCIS) database cohort study.. Arthritis Rheum.

[20] Turhan H, Erbay AR, Yasar AS (2004). Impaired coronary blood flow in patients with metabolic syndrome: Documented by Thrombolysis In Myocardial Infarction (TIMI) frame count method.. Am Heart J.

[21] Taddei S, Virdis A, Ghiadoni L (1998). Vitamin C improves endotheliumdependent vasodilation by restoring nitric oxide activity in essential hypertension.. Circulation.

[22] Panza JA, Quyyumi AA, Brush JE jr, Epstein SE (1990). Abnormal endothelium-dependent vascular relaxation in patients with essential hypertension.. N Engl J Med.

[23] Higashi Y, Sasaki S, Kurisu S (1999). Regular aerobic exercise augments endothelium-dependent vascular relaxation in normotensive as well as hypertensive subjects role of endothelium-derived nitric oxide.. Circulation.

[24] Treasure CB, Klein JL, Vita JA (1993). Hypertension and left ventricular hypertrophy are associated with impaired endothelium-mediated relaxation in human coronary resistance vessels.. Circulation.

[25] Higashi Y, Oshima T, Ozono R (1995). Effects of L-arginine infusion on renal hemodynamics in patients with mild essential hypertension.. Hypertension.

[26] Schiffrin EL, Deng LY (1995). Comparison of effects of angiotensin I-converting enzyme inhibition and β-blockade for 2 years on function of small arteries from hypertensive patients.. Hypertension.

[27] Higashi Y, Nakagawa K, Kimura M (2002). Circadian variation of blood pressure and endothelial function in patients with essential hypertension: a comparison of dippers and non-dippers.. J Am Coll Cardiol.

